# Draft Genome Sequence of the Phenol-Degrading Bacterium *Cupriavidus* sp. Strain P-10, Isolated from Trichloroethene-Contaminated Aquifer Soil

**DOI:** 10.1128/MRA.01009-18

**Published:** 2018-11-08

**Authors:** Kenshi Suzuki, Fatma A. A. Aziz, Masahiro Honjo, Tomoka Nishimura, Kensei Masuda, Ayaka Minoura, Yuki Kudo, Ryota Moriuchi, Hideo Dohra, Yosuke Tashiro, Hiroyuki Futamata

**Affiliations:** a Graduate School of Science and Technology, Shizuoka University, Shizuoka, Japan; b Laboratory of Food Crops, Institute of Tropical Agriculture, Universiti Putra Malaysia, Seri Kembangan, Malaysia; c Graduate School of Integrated Science and Technology, Shizuoka University, Shizuoka, Japan; d Research Institute of Green Science and Technology, Shizuoka University, Shizuoka, Japan; e Department of Biological Science, Graduate School of Integrated Science and Technology, Shizuoka University, Shizuoka, Japan; Indiana University Bloomington

## Abstract

A batch culture was enriched on phenol with trichloroethene-contaminated aquifer soil as an inoculum. Cupriavidus sp. strain P-10 was isolated from the culture using a diluted plating method. Here, we report the draft genome sequence and annotation of strain P-10, which provides insights into the metabolic processes of phenol degradation.

## Abstract (precis)

A batch culture was enriched on phenol with trichloroethene-contaminated aquifer soil as an inoculum. Cupriavidus sp.

## ANNOUNCEMENT

Members of the Cupriavidus genus have been studied as model organisms of heavy metal resistance, synthesis of polyhydroxyalkanoates, and degradation of aromatic compounds for biotechnology applications (1–3). Phenol is a common constituent of effluents derived from various industrial processes, such as polymeric resin production, petroleum refining, and pharmaceutical manufacturing. Since phenol is highly toxic and easily dissolved in water, phenol contamination is a serious problem for human life. The biodegradation of phenol is commonly used to clean up polluted environments; hence, sequencing the genomes of environmental microbes to unveil the metabolic processes of phenol degradation will assist in the construction of effective biodegradation systems (4, 5). Here, we present the genome sequence of Cupriavidus sp. strain P-10, identified as Ralstonia sp. strain P-10 in our previous work, which was isolated from a batch enrichment culture grown on phenol with trichloroethene-contaminated aquifer soil as the inoculum (6).

The genomic DNA of strain P-10 was extracted by a method reported previously (7) and was fragmented using the Covaris acoustic solubilizer. A paired-end library was constructed with the TruSeq DNA PCR-free library preparation kit and was sequenced using the Illumina MiSeq platform (300-bp paired ends) at the Instrumental Research Support Office of the Research Institute of Green Science and Technology at Shizuoka University. The raw read sequences were cleaned up using Trimmomatic v. 0.36 (8) by trimming adapter sequences, low-quality ends, which were defined as having a quality score of less than 15, the last 300 bases, and reads less than 150 bp. The quality-controlled reads were assembled using SPAdes v. 3.10.0 (9), with a default set of k-mer sizes and options (–careful, –only-assembler, and –cov-cutoff auto), and contigs less than 200 bp and less than 10-fold coverage were eliminated. Through this process, 1,801,979 high-quality reads totaling 490 Mb, which corresponds to an approximately 54-fold coverage of the genome, were assembled. The draft genome sequence of strain P-10 has a total length of 8,983,816 bp, including 174 contigs, with an estimated G+C content of 65.3% and an *N*_50_ value of 227,897 bp.

The draft genome was annotated by Rapid Annotations using Subsystem Technology (RAST) (10) and the Microbial Genome Annotation Pipeline (MiGAP) (11). The annotated genome includes 8,184 coding sequences and 66 tRNA sequences. A Southern blot hybridization targeting 16S rRNA genes was conducted to identify the number of 5S-16S-23S rRNA clusters, and five clusters were obtained. The relatedness of Cupriavidus sp. P-10 with other Cupriavidus spp. was assessed by calculating the average nucleotide identity (ANI) using JSpecies (12). The highest ANI at 92% was with Cupriavidus oxalaticus NBRC 13593 (13).

Genes encoding for a multicomponent-type phenol hydroxylase, which is one of the major enzymes related to phenol degradation found in natural environments (14), were found in the genome of strain P-10, as in our previous study (7). The complete set of *meta*-cleavage and *ortho*-cleavage metabolic pathways for catechol via catechol 2,3-dioxygenase and catechol 1,2-dioxygenase (3), respectively, were also found. The gene encoding biphenyl 2,3-dioxygenase, which also converts catechol to 2-hydroxymuconate semialdehyde, was found as part of the benzoate-degrading process (3). This genome information of strain P-10 suggests the presence of multiple phenol metabolic pathways, via *meta*-cleavage or *ortho*-cleavage, which may be exploitable for designing an effective biodegradation system for phenol or other phenolic compounds.

### Data availability.

The raw read sequences have been deposited at DDBJ under the accession no. DRA007291. The draft genome of *Cupriavidus* sp. P-10 has been deposited at DDBJ/GenBank under the accession no. BGPQ01000001 to BGPQ01000174.

## References

[1] Bütof L, Wiesemann N, Herzberg M, Altzschner M, Holleitner A, Reith F, Nies DH 2018 Synergistic gold–copper detoxification at the core of gold biomineralisation in *Cupriavidus metallidurans*. Metallomics 10:278–286. doi:10.1039/C7MT00312A. 29308809

[2] Jiang X, Luo X, Zhou N-Y 2015 Two polyhydroxyalkanoate synthases from distinct classes from the aromatic degrader *Cupriavidus pinatubonensis* JMP134 exhibit the same substrate preference. PLoS One 10:e0142332. doi:10.1371/journal.pone.0142332. 26544851 PMC4636328

[3] Pérez-Pantoja D, De la Iglesia R, Pieper DH, González B 2008 Metabolic reconstruction of aromatic compounds degradation from the genome of the amazing pollutant-degrading bacterium *Cupriavidus necator* JMP134. FEMS Microbiol Rev 32:736–794. doi:10.1111/j.1574-6976.2008.00122.x. 18691224

[4] Suzuki K, Aziz FAA, Inuzuka Y, Tashiro Y, Futamata H 2016 Draft genome sequence of *Pseudomonas* sp. LAB-08 isolated from trichloroethene-contaminated aquifer soil. Genome Announc 4:e00948-16. doi:10.1128/genomeA.00948-16. 27660772 PMC5034123

[5] Azwani F, Suzuki K, Honjyo M, Tashiro Y, Futamata H 2017 Draft genome sequence of *Comamonas testosteroni* R2, consisting of aromatic compound degradation genes for phenol hydroxylase. Genome Announc 5:e00875-17. doi:10.1128/genomeA.00875-17. 28883136 PMC5589530

[6] Futamata H, Harayama S, Watanabe K 2001 Diversity in kinetics of trichloroethylene-degrading activities exhibited by phenol-degrading bacteria. Appl Microbiol Biotechnol 55:248–253. doi:10.1007/s002530000500. 11330722

[7] Futamata H, Harayama S, Watanabe K 2001 Group-specific monitoring of phenol hydroxylase genes for a functional assessment of phenol-stimulated trichloroethylene bioremediation. Appl Environ Microbiol 67:4671–4677. doi:10.1128/AEM.67.10.4671-4677.2001. 11571171 PMC93218

[8] Bolger AM, Lohse M, Usadel B 2014 Trimmomatic: a flexible trimmer for Illumina sequence data. Bioinformatics 30:2114–2120. doi:10.1093/bioinformatics/btu170. 24695404 PMC4103590

[9] Bankevich A, Nurk S, Antipov D, Gurevich AA, Dvorkin M, Kulikov AS, Lesin VM, Nikolenko SI, Pham S, Prjibelski AD, Pyshkin AV, Sirotkin AV, Vyahhi N, Tesler G, Alekseyev MA, Pevzner PA 2012 SPAdes: a new genome assembly algorithm and its applications to single-cell sequencing. J Comput Biol 19:455–477. doi:10.1089/cmb.2012.0021. 22506599 PMC3342519

[10] Aziz RK, Bartels D, Best AA, DeJongh M, Disz T, Edwards RA, Formsma K, Gerdes S, Glass EM, Kubal M, Meyer F, Olsen GJ, Olson R, Osterman AL, Overbeek RA, McNeil LK, Paarmann D, Paczian T, Parrello B, Pusch GD, Reich C, Stevens R, Vassieva O, Vonstein V, Wilke A, Zagnitko O 2008 The RAST server: Rapid Annotations using Subsystems Technology. BMC Genomics 9:75. doi:10.1186/1471-2164-9-75. 18261238 PMC2265698

[11] Sugawara H, Ohyama A, Mori H, Kurokawa K 2009 Microbial genome annotation Pipeline (MiGAP) for diverse users. *In* The 20th International Conference on Genome Informatics (GIW2009) poster and software demonstrations (Yokohama), poster S001-1-2 Imperial College Press, Yokohama, Japan.

[12] Richter M, Rosselló-Móra R 2009 Shifting the genomic gold standard for the prokaryotic species definition. Proc Natl Acad Sci U S A 106:19126–19131. doi:10.1073/pnas.0906412106. 19855009 PMC2776425

[13] Sato Y, Nishihara H, Yoshida M, Watanabe M, Rondal JD, Concepcion RN, Ohta H 2006 *Cupriavidus pinatubonensis* sp. nov. and *Cupriavidus laharis* sp. nov., novel hydrogen-oxidizing, facultatively chemolithothrophic bacteria isolated from volcanic mudflow deposits from Mt. Pinatubo in the Philippines. Int J Syst Evol Microbiol 56:973–978. doi:10.1099/ijs.0.63922-0. 16627640

[14] Perpetuo EA, Marques RCP, Mendes MA, de Lima WC, Menck CFM, do Nascimento CAO 2009 Characterization of the phenol monooxygenase gene from *Chromobacterium violaceum*: potential use for phenol biodegradation. Biotechnol Bioprocess Eng 14:694–701. doi:10.1007/s12257-008-0266-2.

